# Acute Effects of Osteopathic Treatment in Long COVID-19 Patients with Fatigue Symptoms: A Randomized, Controlled Trial

**DOI:** 10.3390/jcm14176066

**Published:** 2025-08-27

**Authors:** Ulrich M. Zissler, Tino Poehlmann, Rainer Gloeckl, Sami Ibrahim, Kerstin Klupsch, Tessa Schneeberger, Inga Jarosch, Andreas Rembert Koczulla

**Affiliations:** 1 Technology Transfer Center (TTZ) for Building Biology, Airway and Indoor Health, University of Applied Sciences Rosenheim, 83395 Freilassing, Germany; 2 Department of Medicine, Pulmonary and Critical Care Medicine, University Medical Center Giessen and Marburg, Philipps University Marburg, Member of the German Center for Lung Research (DZL), Universities of Giessen and Marburg Lung Center (UGMLC), 35043 Marburg, Germany; 3 Department of Pediatrics, Klinikum Rechts der Isar, TUM School of Medicine and Health, Technical University of Munich (TUM), 80804 Munich, Germany; 4 Vienna School of Osteopathy, 1150 Vienna, Austria; 5 Institute for Pulmonary Rehabilitation Research, Schoen Klinik Berchtesgadener Land, 83471 Schoenau am Koenigssee, Germany; rgloeckl@schoen-klinik.de (R.G.); tschneeberger@schoen-klinik.de (T.S.); ijarosch@schoen-klinik.de (I.J.); rkoczulla@schoen-klinik.de (A.R.K.); 6 Department of Pulmonary Rehabilitation, German Center for Lung Research (DZL), University Medical Center Giessen and Marburg, Philipps-University Marburg (UMR), 35043 Marburg, Germany; 7 IU Internationale Hochschule, 81673 Munich, Germany; 8 Teaching Hospital, Paracelsus Medical University Salzburg, 5020 Salzburg, Austria

**Keywords:** post COVID, fatigue symptoms, osteopathic manipulative treatment, osteopathy, heart rate variability, autonomic nervous system

## Abstract

**Background**: Persistent fatigue is among the most commonly reported symptoms in patients suffering from post-acute sequelae of SARS-CoV-2 infection (long COVID). Autonomic dysfunction, measurable via heart rate variability, has been implicated as a contributing factor. Osteopathic manipulative treatment is a manual therapeutic approach that targets autonomic balance and may offer a novel intervention for long COVID-related fatigue. **Methods**: In this single-blind, randomized controlled trial, 42 participants (mean age 51 ± 11 years; fatigue severity score: 31 ± 5 points) with long COVID and persistent fatigue ≥12 weeks post-infection were allocated to either a 45 min standardized osteopathic treatment (*n* = 21) or a sham-treatment group (*n* = 21). Heart rate variability was assessed using a 10 min resting electrocardiogram before intervention, immediately after, and again 48 h later. The analysis of heart rate variability encompassed time-domain indices, including the root mean square of successive differences, the standard deviation of normal-to-normal intervals, mean heart rate, and mean RR interval. Additionally, frequency-domain measures such as low-frequency, high-frequency, total power, and the LF/HF ratio were considered. **Results**: The osteopathy group showed a statistically significant increase in root mean square of successive differences post-treatment (*p* < 0.01), accompanied by a decrease in the stress index (*p* < 0.05) and an increase in the mean of the standard deviations of RR intervals (*p* < 0.05). Significant between-group differences were observed for mean heart rate and mean of RR intervals (*p* < 0.05). Frequency-domain measures also improved significantly from baseline in the intervention group. Outlier patterns suggest potential subgroup effects, possibly due to underlying dysautonomia. **Conclusions**: A single session of osteopathic treatment significantly enhanced short-term heart rate variability in long COVID patients with fatigue. These findings highlight the potential role of manual autonomic modulation as a supportive therapy in long COVID management. Further research is needed to assess the long-term effects and optimal treatment frequency of osteopathic manipulative treatment in this population.

## 1. Introduction

Since the onset of the COVID-19 pandemic, an estimated 47% of the German population has been infected with SARS-CoV-2, with a case fatality rate of 0.47% [1]. While most patients recover from the acute phase, a substantial proportion continues to experience persistent symptoms, a condition now recognized as long COVID or post-COVID-19 syndrome. A large-scale prospective study reported that 13.3% of participants had symptoms lasting ≥28 days, 4.5% ≥8 weeks, and 2.3% ≥12 weeks after the initial infection [2]. Commonly reported symptoms include fatigue, dyspnea, musculoskeletal pain, and insomnia [3]. However, standard clinical investigations often fail to identify specific pathophysiological correlates [4]. Fatigue, in particular, is a leading complaint among long COVID patients and shares clinical features with post-infectious fatigue syndromes triggered by pathogens such as Epstein-Barr virus, influenza, enteroviruses, and intracellular bacteria [5,6]. Epidemiological data highlight the relevance of this issue: in a representative German population sample, the prevalence of severe fatigue symptoms was 14.5%, with higher rates among women and individuals with chronic illness or low socioeconomic status [7]. These findings underscore the urgent need for effective, low-risk treatment options for fatigue-related conditions. A growing body of evidence suggests that autonomic nervous system (ANS) dysfunction may contribute to the pathogenesis of long COVID symptoms. For instance, reduced parasympathetic activity and sinus tachycardia have been observed in 24 h heart rate variability (HRV) assessments of long COVID patients [8,9]. HRV is a well-established, non-invasive measure of autonomic balance and has emerged as a promising biomarker for dysautonomia and stress-related disorders. Osteopathic manipulative treatment (OMT) is a core component of osteopathic medicine, a holistic and patient-centered form of manual therapy that was developed in the late 19th century by the American physician Andrew Taylor Still. This approach is based on the idea that a person’s structure and function are connected and that the body has self-regulatory mechanisms for healing. OMT encompasses various hands-on techniques that aim to improve physiological function by addressing somatic dysfunctions in the musculoskeletal, visceral, and cranial systems. A central tenet of osteopathy is enhancing autonomic regulation by manipulating fascia, joints, and soft tissues. OMT has been shown to have the ability to modulate the ANS and improve HRV parameters [10,11]. Recent studies suggest that OMT can increase parasympathetic activity—reflected in improvements in high-frequency (HF) power, RMSSD, and SDNN—and reduce sympathetic dominance, as indicated by decreased LF/HF ratios [12]. Given these physiological effects, OMT may offer a supportive therapeutic option for individuals with long COVID-related fatigue. However, empirical evidence for its efficacy in this context remains limited. The primary objective of this randomized, controlled, proof-of-concept trial was to evaluate whether OMT could induce measurable changes in HRV among long-haul COVID-19 patients with fatigue symptoms, using a sham-controlled design. In addition to HRV, the secondary objectives included assessing self-reported fatigue levels and subjective well-being before and after the intervention. We hypothesized that participants receiving OMT would demonstrate significantly greater improvements in HRV parameters, particularly increased parasympathetic activity, such as HF, RMSSD, and SDNN, but also decreased sympathetic dominance (LF/HF ratio). Furthermore, we expected OMT to be associated with greater reductions in fatigue severity and improved self-perceived health status.

## 2. Methods

### 2.1. Study Design

This single-center, randomized, controlled trial (RCT) was conducted in accordance with the ICH-GCP guidelines and the Declaration of Helsinki. Ethical approval was obtained from the Ethics Committee of the Department of Human Medicine at Philipps University Marburg (Ref. No. 23-08 BO). The study was prospectively registered in the German Register of Clinical Trials (DRKS-ID: DRKS00030829) and is listed on the WHO International Clinical Trials Registry Platform (ICTRP). Participant recruitment and data collection, including all HRV measurement time points, were conducted under supervision between June and November 2023 at the Schoen Klinik Berchtesgadener Land, Schoenau am Koenigssee, Germany. All participants provided written informed consent. Randomization was performed by a staff member not involved in the intervention or data analysis. The randomization process was executed through the utilization of sealed envelopes, which were arranged into three blocks of ten and two blocks of six numbers, without the predetermined block characteristics. An independent and blinded third party was designated to reveal the group assignment upon request and link it to the respective study identification code. Outcome assessors and data analysts were blinded to group allocation.

### 2.2. Study Procedures

**Figure 1:**
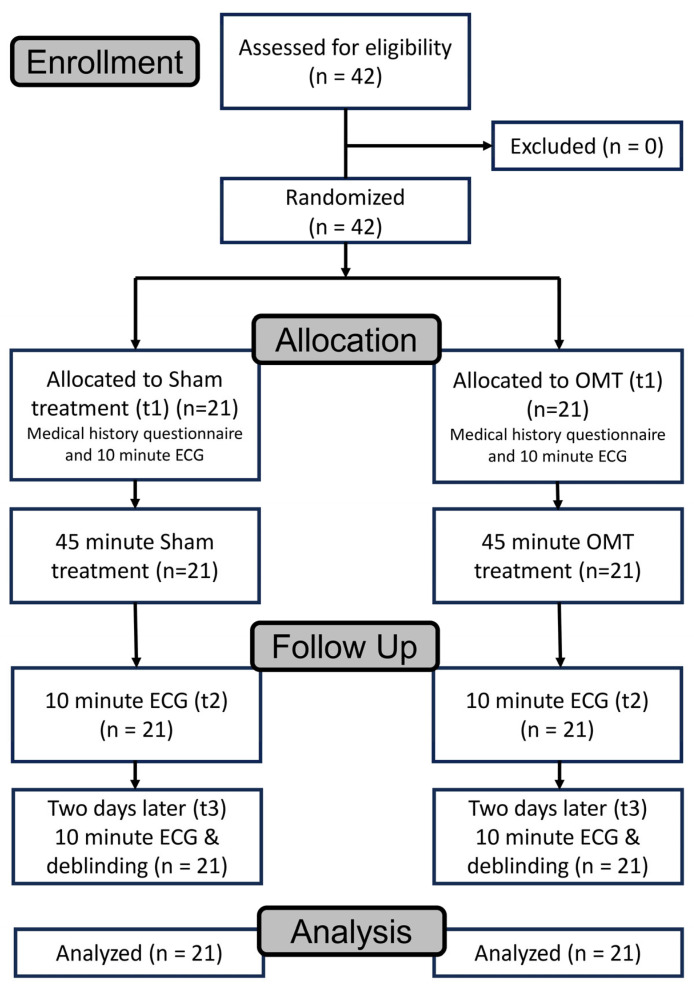
Consort flow chart.

The study comprised three time points: baseline (T1), immediately after the intervention (T2), and 48 h follow-up (T3; Figure 1). Prior to inclusion, each participant underwent a standardized medical evaluation to confirm a diagnosis of long COVID-19 syndrome according to Koczulla et al. (2021) [4]. Participants completed a medical history questionnaire, the Fatigue Assessment Scale (FAS), and a subjective dyspnea questionnaire assessing exercise-induced breathlessness on a scale from 0 (no impairment) to 10 (unbearable). To assess the integrity of blinding, participants completed a de-blinding questionnaire at follow-up, which asked whether they believed they had received an actual treatment or placebo, based on prior protocols [13,14].

### 2.3. Participants

A total of 42 participants were included (Figure 1). No subjects were excluded during enrollment. Inclusion criteria were: age ≥ 18 years; documented SARS-CoV-2 infection with persistent symptoms for ≥4 weeks; and an FAS score ≥22. Participants were excluded if they had previously received osteopathic treatment for fatigue or long COVID symptoms. Further exclusion criteria included current alcohol or drug abuse (due to its influence on HRV) and first-trimester pregnancy due to limited data on intervention safety in this group [15]. The study included subjects who were over 18 years old at the time of data collection. The patients had previously recovered from an acute SARS-CoV-2 infection and were in the post-acute phase at least 4 weeks afterward, as defined in the NICE guidelines [3]. Since low HRV values are typically observed in patients with fatigue syndrome [16], patients experiencing fatigue were included to ensure baseline value homogeneity. Fatigue is the most common symptom in long COVID, accounting for 58% of patients [17]. The FAS was used to confirm the presence of fatigue, and patients with a cutoff score of 22 or higher were included in the study. All patients underwent a conventional medical differential diagnosis at the start of their rehabilitation to confirm the LPCS diagnosis [4,18]. To best ensure blinding of the control group receiving the sham treatment, subjects who had not previously received osteopathic treatment for this condition were included. Subjects with a history of alcohol or drug abuse were excluded. Such abuse can reduce HRV in the short and long term, which could influence the analysis [15]. Pregnant women in the first trimester were also excluded.

### 2.4. Osteopathic Treatment Intervention

#### 2.4.1. Osteopathic Treatment Group

Participants in the intervention group received a single 45 min session of osteopathic treatment. A standardized assessment was performed in standing, sitting, and supine positions using the Zink test to identify somatic dysfunctions (Figure 1). Diagnoses were based on tissue texture abnormalities, asymmetry, range of motion restrictions, and tenderness. Treatment was individualized according to osteopathic findings and applied using techniques deemed appropriate by the therapist. Interventions were performed by two certified osteopaths with extensive clinical experience.

#### 2.4.2. Sham-Treatment of Control Group

Control group participants received a 45 min sham treatment designed to mimic the structure of the OMT session (Figure 1). Practitioners placed their hands on various body regions without applying therapeutic manipulations and silently counted backward to simulate treatment duration and engagement [12,19]. No verbal feedback or clinical findings were shared during or after the sessions. This approach has been validated in previous studies examining autonomic nervous system outcomes [13,14].

### 2.5. Outcome Measures

Heart rate variability (HRV) was recorded for 10 min using the Faros 180 ECG device (Bittium GmbH, Oulu, Finland) at each time point. Participants were instructed to remain awake, breathe calmly (approx. 9–20 breaths/min), and avoid physical activity before measurement (Figure 1). Data were analyzed using Kubios HRV software 4.0.2 (Kubios Oy, Kuopio, Finland), which supports multiple ECG input formats and computes both linear and non-linear HRV parameters [20]. Measured parameters included time-domain metrics (RMSSD, mean RR, mean HR, SDNN, pNN50, NN50), frequency-domain metrics (LF, HF, LF/HF ratio), and non-linear dynamics (Poincaré plot). The Baevsky Stress Index, representing sympathetic nervous system activity, was also calculated based on RR interval variability [21].

### 2.6. Data Analysis

Sample size was estimated using G*Power software 3.1.9.7 [22]. Based on a similar study [11], we used the smallest reported effect size (ƞp^2^ = 0.117), an alpha error level of 0.05, and a power of 0.8 to be on the safe side. Using these parameters for the F-test, we obtained a total sample size of *n* = 42. This corresponds to 21 individuals in each group. Normality was assessed using the Shapiro–Wilk test; homogeneity of variances via Levene’s test. For repeated measures, a robust mixed ANOVA was conducted to evaluate interaction effects (group × time). In addition, a multistep, estimation-oriented approach was applied to assess intragroup and intergroup effects across time points. For each variable, descriptive statistics and standardized effect sizes (Hedges’ g) were calculated, including 95% confidence intervals (CI) via bias-corrected bootstrapping (1000 resamples); for group differences, small, medium, and large effect sizes correspond to 0.1, 0.4, and 0.8, respectively [23]. This enabled a reliable interpretation of temporal trends and effect magnitudes, accounting for deviations from normality and unequal variances. To validate findings and estimate individual variability over time, a simulation-based bootstrap procedure (B = 1000) was used to generate empirical confidence envelopes for mean HRV trajectories. This approach follows APA recommendations [24,25] by prioritizing effect size estimation over sole reliance on *p*-values [26]. Baseline comparability between the osteopathy and sham groups was assessed using appropriate tests. These tests confirmed no significant baseline differences between groups across demographic and clinical parameters (see Table 1).

## 3. Results

### 3.1. Symptoms Associated with Long COVID and Fatigue Symptoms

**Figure 2:**
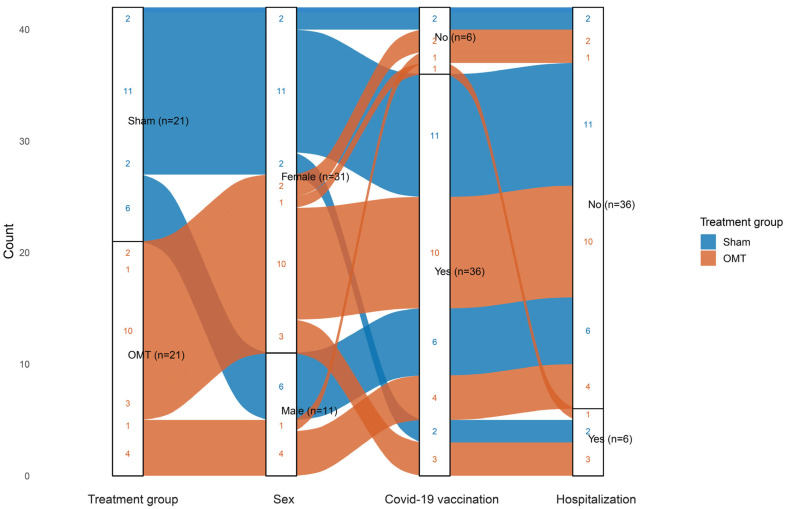
Sankey diagram depicting the distribution and intersection of treatment group (osteopathic manual treatment [OMT] vs. Sham), sex, COVID-19 vaccination status, and hospitalization among study participants (*n* = 42). Flow width reflects the number of individuals per category combination.

A total of 42 participants were included in the study, with ages ranging from 23 to 75 years (Table 1). The mean age was 50.1 ± 11.7 years in the sham group and 51.0 ± 12.5 years in the osteopathy group. Overall, 26% (*n* = 11) of participants were male and 74% (*n* = 31) female. A Sankey diagram (Figure 2) illustrates the distribution and overlap of treatment group (OMT vs. sham), sex, vaccination status, and prior COVID-19-related hospitalization. The width of each stream corresponds to the number of individuals in the respective category combination. The mean time from initial SARS-CoV-2 infection to the study intervention was 15.1 ± 7.5 months. In the sham group, this interval was 12.5 ± 1.2 months, compared to 17.2 ± 1.9 months in the OMT group. Most participants (*n* = 36) had been vaccinated prior to their acute infection, with 19 in the sham group and 17 in the OMT group. Detailed information on acute symptom profiles is provided in Table 1.

### 3.2. Stress and Heart Rate Changes in OMT Treatment

**Figure 3:**
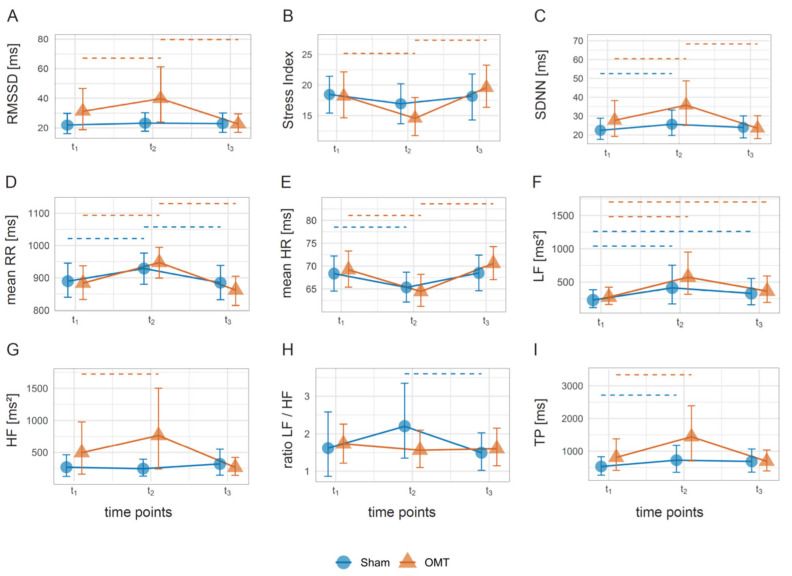
Trajectories of significant within-subject differences (Hedges’ *g*) visualized by dashed lines comparing three time points (t1, t2, t3) between participants in the osteopathy group (blue) and sham group (yellow). (**A**) Root mean square of successive differences (RMSSD); (**B**) the Baevsky Stress Index; (**C**) Standard deviation of normal-to-normal interbeat intervals (SDNN); (**D**) average time between heartbeats for a reading (mean RR); (**E**) mean heart rate (mean HR); (**F**) low frequency power (LF); (**G**) high frequency power; (**H**) ration LF/HF); (**I**) total power.

Compared to the control group receiving sham treatment (placebo control), the osteopathy group exhibited consistent and temporally structured changes across key heart rate variability (HRV) indices. RMSSD significantly increased from t_1_ (mean: 17.8 ms ± 11.2, *n* = 15) to t_2_ (mean: 21.6 ms ± 9.3; g = 0.82, 95% CI [0.19, 1.40]) and declined by t_3_ (mean: 17.4 ms ± 7.8; g = −0.55, 95% CI [−0.96, 0.18]; Figure 3A). A similar pattern was observed in the stress index, which initially decreased from t_1_ (mean: 16.2 ± 6.2, *n* = 18) to t_2_ (mean: 12.8 ± 5.0; g = −1.02, 95% CI [−1.33, −0.59]) and subsequently increased again at t_3_ (mean: 18.0 ± 6.8; g = 0.94, 95% CI [0.56, 1.34]; Figure 3B). The SDNN increased significantly from t_1_ (mean: 19.6 ms ± 8.6, *n* = 17) to t_2_ (mean: 26.0 ms ± 12.5; g = 0.80, 95% CI [0.38, 1.18]) and declined at t_3_ (mean: 21.4 ms ± 10.3; g = −0.51, 95% CI [−0.92, 0.01]; Figure 3C).

Mean RR intervals increased markedly between t_1_ (mean: 869 ms ± 104, *n* = 19) and t_2_ (mean: 934 ms ± 102; g = 1.01, 95% CI [0.43, 1.61]) and returned to lower values by t_3_ (mean: 857 ms ± 104; g = −1.04, 95% CI [−1.56, −0.52]; Figure 3D). The inverse pattern emerged for mean HR, which decreased from t_1_ (mean: 67.3 bpm ± 7.8, *n* = 18) to t_2_ (mean: 62.3 bpm ± 5.9; g = −1.10, 95% CI [−1.62, −0.48]) and increased again at t_3_ (mean: 68.8 bpm ± 7.1; g = 1.03, 95% CI [0.53,1.57]; Figure 3E).

In the frequency domain, low-frequency power (LF) increased significantly from t_1_ (mean: 206 ms^2^ ± 153, *n* = 17) to t_2_ (mean: 329 ms^2^ ± 277; g = 0.53, 95% CI [0.07, 0.86]) and remained stable by t_3_ (mean: 247 ms^2^ ± 217; Figure 3F). High-frequency power (HF), as an index of parasympathetic tone, rose from t_1_ (mean: 146 ms^2^ ± 207, *n* = 14) to t_2_ (mean: 213 ms^2^ ± 202; g = 0.48, 95% CI [−0.14, 0.99]) and dropped at t_3_ (mean: 119 ms^2^ ± 91; g = −0.59, 95% CI [−0.95,−0.27]; Figure 3G).

The LF/HF ratio remained statistically unchanged across all time points within the osteopathy group. In contrast, the control group exhibited a moderate decrease in this ratio between t_2_ (mean: 2.05 ± 0.53) and t_3_ (mean: 1.09 ± 0.28; g = −0.53, 95% CI [−0.83, −0.04]), as well as a significant decline in mean RR intervals (g = −0.79, 95% CI [−1.43, −0.03]) in the same interval (Figure 3H). Total power (TP) rose from t_1_ (mean: 487 ms^2^ ± 457, *n* = 18) to t_2_ (mean: 822 ms^2^ ± 773; g = 0.62, 95% CI [0.35, 0.89]) but did not increase further at t_3_ (mean: 561 ms^2^ ± 534; g = −0.36, 95% CI [−0.74, 0.12]; Figure 3I). All other metrics in the sham group remained largely stable.

**Figure 4:**
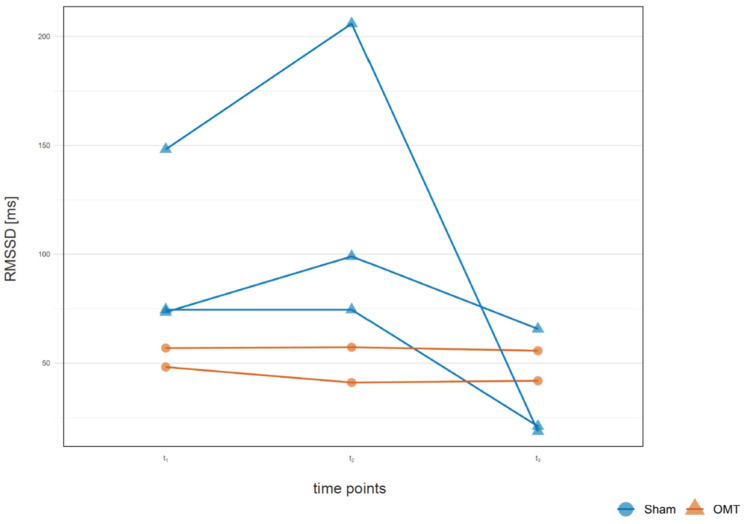
Comparison of time progression (t_1_, t_2_, t_3_) for root mean square of successive differences (*RMSSD*) in five participants in the osteopathy group (blue) and sham group (orange), whose average HRV was significantly above the normal values according to previous publications.

Five outliers were identified in the dataset, four of them in the osteopathy group, and two in the sham group (Figure 4; Table 2). The osteopathic group showed an increase in RMSSD values at t_2_, followed by a significant decrease from t_2_ to t_3_. In the sham group, on the other hand, the values hardly changed. Due to the small sample size, no effect sizes were calculated.

At the end of the study, all participants completed a de-blinding questionnaire. In the sham group, 13 participants correctly assumed that they were in this group, whereas eight thought that they had received osteopathic treatment. This results in a percentage ratio of 61.9% to 38.1%. In the de-blinding questionnaire, respondents were asked how strongly they believed that treatment had taken place and were rated on an NRS of 0–10. The participants who had seen themselves in the osteopathy group gave a mean value of 6.7 ± 2.6, and those who had seen themselves in the placebo group 5.8 ± 2.9.

## 4. Discussion

Persistent symptoms beyond the acute phase of SARS-CoV-2 infection—lasting weeks to months—are referred to as long COVID-19 syndrome [3,4,27]. In our study cohort, participants presented with fatigue-related symptoms and autonomic dysfunction, paralleling findings observed in myalgic encephalomyelitis/chronic fatigue syndrome (ME/CFS). In ME/CFS, significantly reduced HRV parameters—such as mean RR, SDNN, RMSSD, and pNN50—have been reported, reflecting reduced vagal tone and increased sympathetic activity [16]. Similarly, frequency domain analyses in ME/CFS revealed decreased LF and HF power and an elevated LF/HF ratio, indicating autonomic imbalance. Osteopathic manual treatment (OMT) has been suggested as a potentially beneficial intervention for patients with fatigue syndromes, including ME/CFS [28] and, more recently, for those with long COVID [29]. In the present study, we observed transient yet consistent changes in HRV following OMT: RMSSD and SDNN significantly increased, and the stress index declined shortly after treatment (t_2_), indicating acute improvements in parasympathetic activity. These changes were reversed by the follow-up time point (t_3_), consistent with prior observations of short-term autonomic modulation after therapeutic interventions [30]. This temporal pattern aligns with previous studies: in COVID-19 survivors, RMSSD was observed to increase during early recovery, and similar post-treatment changes have been reported in ME/CFS [31]. However, HRV findings in post-COVID-19 cohorts have been inconsistent across studies [32], with reports of both increased and decreased HRV values [8,33], likely due to differences in timing, symptom severity, and measurement protocols. The observed acute HRV improvement after OMT suggests that the intervention temporarily modulates autonomic tone—potentially via vagal activation—without inducing long-lasting effects after a single session. This is supported by changes in time and frequency domain parameters, including RMSSD, SDNN, mean RR, HF power, and total power, which peaked post-treatment and returned to baseline within 48 h. Notably, stress-related markers (e.g., mean HR, stress index, LF/HF ratio) also showed a reversal between t_2_ and t_3_, indicating a return to sympathetic predominance. Importantly, the sham group showed no comparable changes in HRV parameters, supporting the hypothesis that the effects observed in the OMT group were not due to placebo or expectation alone. This is in line with earlier findings indicating the potential of manual therapies to induce acute autonomic modulation [10,34]. Nevertheless, nonspecific treatment effects such as patient expectations, therapeutic touch, and the general experience of attentive care may have contributed to the observed responses. The impact of placebo effects in manual therapy research has been thoroughly documented, particularly in conditions such as fatigue, where subjective perceptions play a pivotal role in outcome reporting [35,36,37]. Despite the meticulous design of our sham intervention to control for these factors, the possibility of residual expectancy effects cannot be entirely discounted, particularly in light of the open nature of the patient-therapist interaction. The concept of therapeutic touch has been demonstrated to impact autonomic responses, potentially through mechanisms involving affective touch, interoception, and oxytocin release [38]. These aspects are not exclusive to OMT and could potentially partially explain short-term changes in HRV. Five participants in our study exhibited unusually high RMSSD values, deviating from the expected profile of fatigued patients, where RMSSD is typically reduced [16]. Interestingly, 75% of these outliers had been hospitalized during the acute phase of COVID-19, consistent with reports linking post-hospitalization to elevated HRV [32]. However, the possibility that psychiatric comorbidities, such as anxiety disorders—which can paradoxically increase HRV [39]—contributed to these findings must be considered. The use of standardized psychometric assessments in future studies is, therefore, recommended. While the presence of large standard deviations in HRV parameters, such as RMSSD, LF, and HF, underscores high interindividual variability [32,40], the consistent group-level patterns and medium-to-large effect sizes support the reliability of the observed responses to OMT. Factors such as breathing rate, stress perception, and baseline autonomic tone may modulate individual responses [41].

These findings emphasize the importance of person-centered analytic strategies—such as responder classification or latent class modeling—to identify subgroups that benefit most from OMT. Such approaches could enhance therapeutic precision in future studies [42]. It is crucial to consider the study as a pilot one. While the effects of one OMT session appear promising, the results’ limited applicability is shown by the small sample size and brief follow-up period. The observed changes are too temporary to draw definitive therapeutic conclusions yet; OMT might help in the long-term effects of post-COVID syndrome when combined with other treatments. Research is needed on OMT’s role in this context. Previous studies on similar approaches for post-viral syndromes have shown mixed results, so more research is necessary to clarify OMT’s role in broader therapeutic settings [43]. However, the study is not without limitations. The most critical are the small sample size, the absence of long-term follow-up, and the exclusive focus on HRV as an outcome measure. Moreover, the observed return of HRV parameters to baseline within 2 days limits conclusions about sustained therapeutic effects. Self-reported fatigue severity was reduced, but the minimal clinically important difference (MCID) for the Fatigue Assessment Scale (FAS) has not been established in long-COVID populations. The clinical relevance of these changes is unclear, so they should be interpreted with caution. Future studies should determine the MCID for FAS in this context to better interpret therapeutic impact. Future research should involve larger cohorts, repeated treatment sessions, and multimodal outcomes (e.g., fatigue scales, quality of life measures) over extended periods. This study is the first to systematically evaluate the effects of OMT in long COVID-19 patients using robust HRV analysis in a controlled trial setting. The results indicate that OMT can transiently enhance parasympathetic tone and modulate autonomic function in affected individuals. Although the effects appear short-lived, they suggest a therapeutic window for intervention. Nonetheless, it must be noted that these effects were short-lived and should be interpreted as preliminary rather than conclusive evidence for therapeutic efficacy. In the broader context, the rhythmic nature of HRV in relation to biological cycles—such as sleep, activity, and hormonal rhythms—aligns conceptually with the rhythmic principles of osteopathy. Techniques that aim to synchronize craniosacral and tissue rhythms could offer new avenues for monitoring and optimizing treatment outcomes through long-term HRV assessment.

In conclusion, while this study demonstrates that OMT can induce short-term autonomic improvements in patients with long COVID-19, the return to baseline by t_3_ highlights the need for repeated treatments and longitudinal research. Future studies should evaluate the durability of these effects and assess whether sustained OMT interventions can contribute to long-term recovery in individuals with post-viral autonomic dysfunction.

## Figures and Tables

**Table 1:** Patient Characteristics.

	Osteopathic Manipulative Treatment	Sham Treatment	*p*-Value
Number of Participants [*n*]	21	21	n.d.
Age [years]	51.0 ± 12.5	50.1 ± 11.7	0.81 ^(a)^
Sex [female/male]	16/5	15/6	>0.99 ^(b)^
FAS	31.2 ± 5.6	34.4 ± 5.6	0.07 ^(a)^
Vaccinated before infection [*n*]	17 (41%)	19 (45%)	0.66 ^(c)^
Duration since SARS-CoV-2 infection [d]	543.7 ± 65.0	433.1 ± 61.9	0.23
Health issues (in acute phase)			
Common cold w/o fever	6 (14%)	3 (7%)	0.45 ^(c)^
Common cold with fever	11 (26%)	13 (31%)	0.76 ^(c)^
Shortness of Breath and Cough	14 (33%)	15 (36%)	>0.99 ^(b)^
Hospitalization	4 (10%)	2 (5%)	0.66 ^(b)^
Admission to intensive care unit (ICU)	1 (3%)	0 (0%)	>0.99 ^(b)^
Fatigue or weakness (scale score)	7.1 ± 2.5	8.1 ± 1.8	0.21 ^(a)^
Joint and muscle pain (scale score)	5.7 ± 3.3	5.6 ± 3.4	>0.99 ^(a)^
Sleep disturbances (scale score)	4.9 ± 3.5	6.6 ± 3.3	
Anxiety	1.6 ± 2.0	3.4 ± 3.4	0.05 ^(a)^
Loss of self-control	1.7 ± 2.7	4.7 ± 3.1	0.02 ^(a)^
Lack of drive, interest, or feeling lonely (scale score)	3.6 ± 3.7	4.7 ± 3.1	0.33 ^(a)^
Impaired balance or dizziness (scale score)	3.5 ± 2.9	5.0 ± 3.9	0.17 ^(a)^
Loss of smell and taste (scale score)	0.9 ± 2.1	1.5 ± 2.5	0.42 ^(a)^

^(a)^ ANOVA; ^(b)^ Fisher’s Exact Test; ^(c)^ Chi-Square-Test.

**Table 2:** Outliers in comparison to entire study population.

	Entire Study Population	Outlier
Number of Participants [*n*]	29	6
Range of Age [years]	29–75	32–48
RMSSD [ms]	20.5	88.3
SDNN [ms]	21.5	67.3
FAS	33	29

## Data Availability

All data are available upon request from the authors.

## Abbreviations

ANS	autonomic nervous system
FAS	fatigue assessment scale
HF	high frequency
HRV	heart rate variability
ICTRP	WHO international Clinical Trials Registry Platform
LF	low frequency
mHR	mean Heart Rate
MCID	minimal clinically important difference
mRR	mean RR intervals
OMT	Osteopathic manipulative treatment
RCT	Randomized, controlled trial
RMSSD	Root mean square of successive differences
SDNN	Mean of the standard deviations of RR intervals
SI	Baevsky’s Stress index
TP	Total power of HRV
